# Supplementary material for: Structured multimaterial filaments for 3D printing of optoelectronics
Source: Nat Commun. 2019 Sep 5;10:4010. doi: 10.1038/s41467-019-11986-0 (PMC6728390; doi:10.1038/s41467-019-11986-0)
Supplement: Supplementary file 1 — Supplementary Information [file 41467_2019_11986_MOESM1_ESM.pdf]

Supplementary Information

Structured multimaterial filaments for 3D printing of optoelectronics

Loke *et al.* (2019)

Supplementary Figures

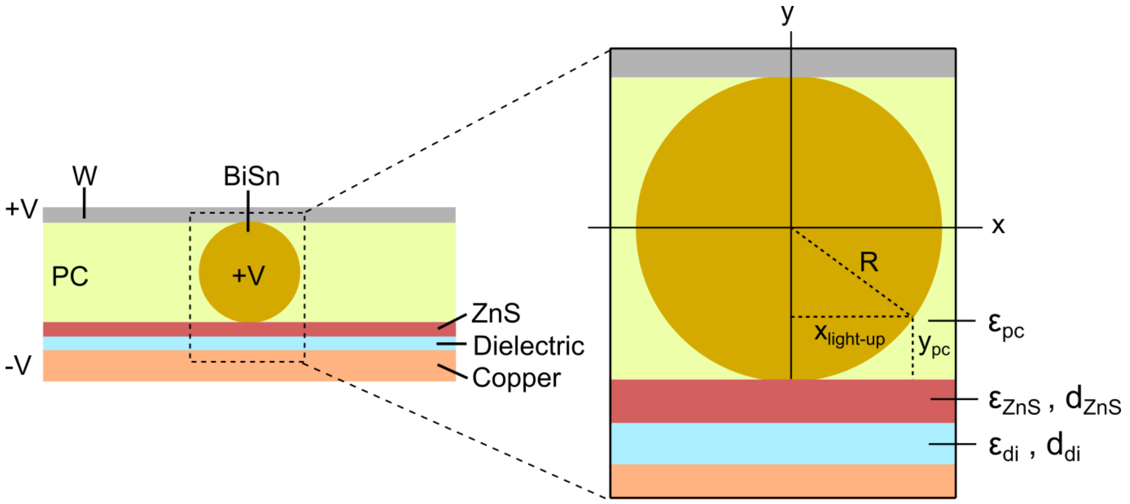

Supplementary Figure 1: Schematic of a pixel sphere interfacing with W and ZnS within the filament. In this schematic, the corresponding dielectric and distance parameters of the different materials are highlighted. This schematic is used in particular for the derivation of the relationship between width of light-emission and sphere size in Supplementary Note 1.

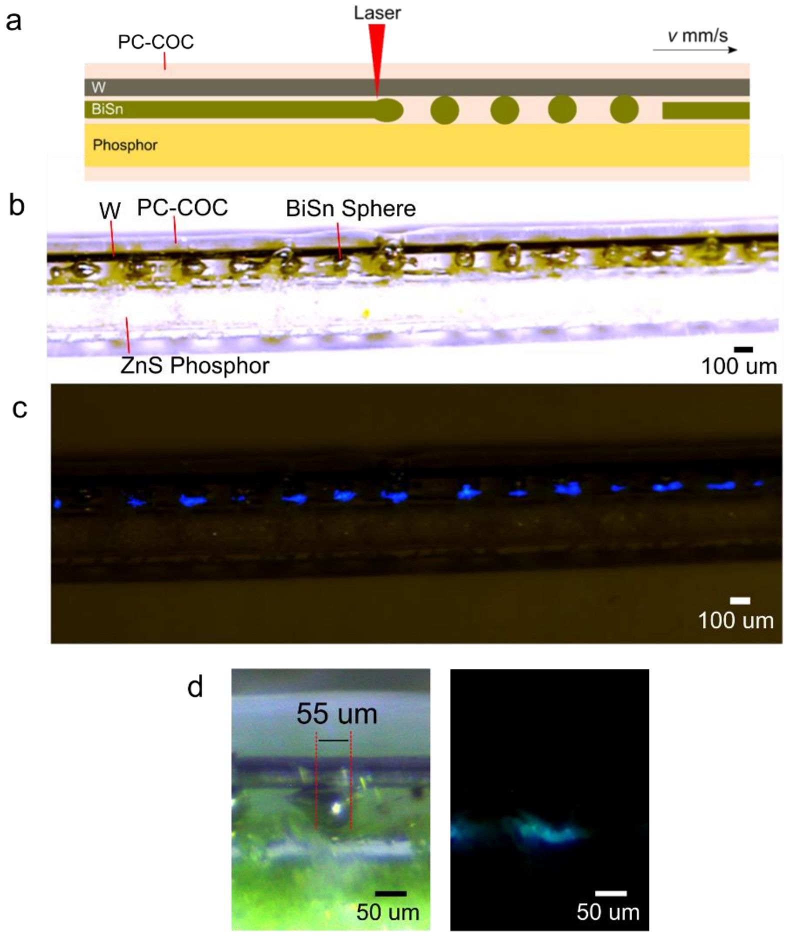

Supplementary Figure 2: Generation of large number of pixels. (a) Schematic of laser-induced capillary breakup of the BiSn core into spheres by translating the filament. Transparent to PC and COC, the 808 nm laser spot can focused directly onto the BiSn core. **(b)** Optical micrographs of large number of pixel-spheres, with the corresponding **(c)** Blue light-up spots at sphere locations. The average pixel density measured here is 4.21 pixels per mm or 107 pixels-per-inch (ppi). **(d)** Capillary formation of a 55 um sphere with its corresponding lighted-up spot when voltage is turned on.

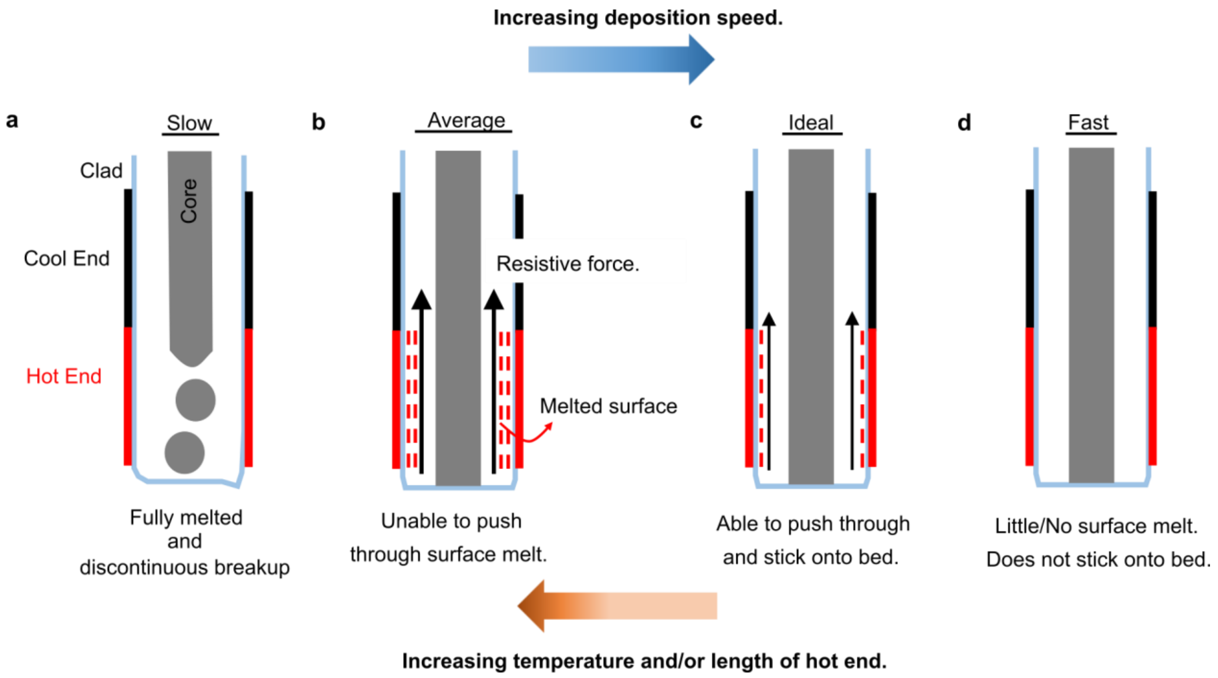

Supplementary Figure 3: Effects of different parameters on microstructures and print quality. (a) At slow deposition speed, high temperature or long hot end, heat will diffuse into the core, resulting in complete melting, intermixing or capillary breakup of the core materials within the cladding material. This prevents control over the shapes and interfaces of the filament microstructure. Note that the core here may refer to 1 or more materials. **(b)** With slightly faster deposition speed, lower temperature or shorter hot end, the filament may still not be able to be smoothly extruded from the nozzle. We find that the heated filament faces surface resistance when the heated surface melts and wets, and later adheres to the walls of the heated channel. This may thus result in jamming of the filament at the feeder. In this regime, with longer channel length of the hot end, the heated filament faces higher surface resistance as a larger surface area of the surface melt adheres to the heated channel walls, consequently causing the feeding filament to remain stuck in the channel of the hot end. There is thus a required minimum feeding speed of the filament through the heated nozzle, in order to overcome this surface resistance acting on the feeding filament. **(c)** In the ideal scenario, there is a right combination of the input deposition speed, length and temperature of the hot end. The filament should be able to extrude smoothly from the nozzle, form curves and bends, as well as adhere and fuse well to the existing printing layers, while preserving the microstructures. **(d)** For low temperature, short hot end or fast deposition speed, even though the structure of the core will be well-preserved, there is a lack of surface melt (adhesive promoter) for the extruded filament to adhere well to the printing bed and existing layers.

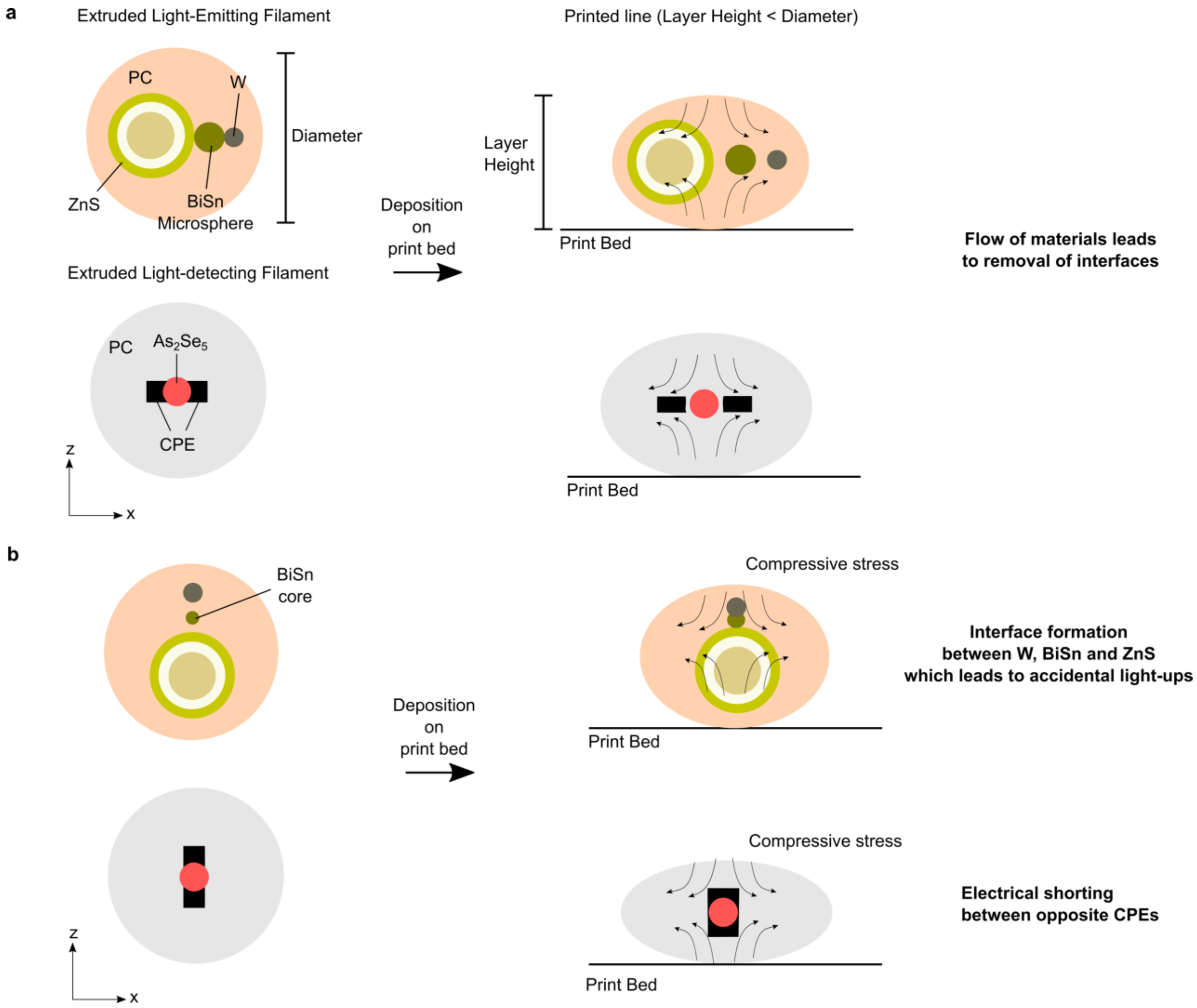

Supplementary Figure 4: Manipulating interfaces of microstructures upon deposition on print bed. (a) Deposition of the extruded filament with the functional domain aligned along the x direction. Due to the flow of materials during deposition and wetting of the polymeric matrix, materials are pulled away from each other in the x-direction, leading to removal of interfaces. **(b)** Deposition of the extruded filament with the functional domain aligned along the z direction. Instead of interface-removal, new interfaces can be formed if the filament is oriented in this geometry when printing. While forming interfaces is useful in producing functional microstructure, it may also lead to undesired interface formations such as accidental light-ups in the printed light-emitting filament or electrical shorting in the printed light-detecting filament.

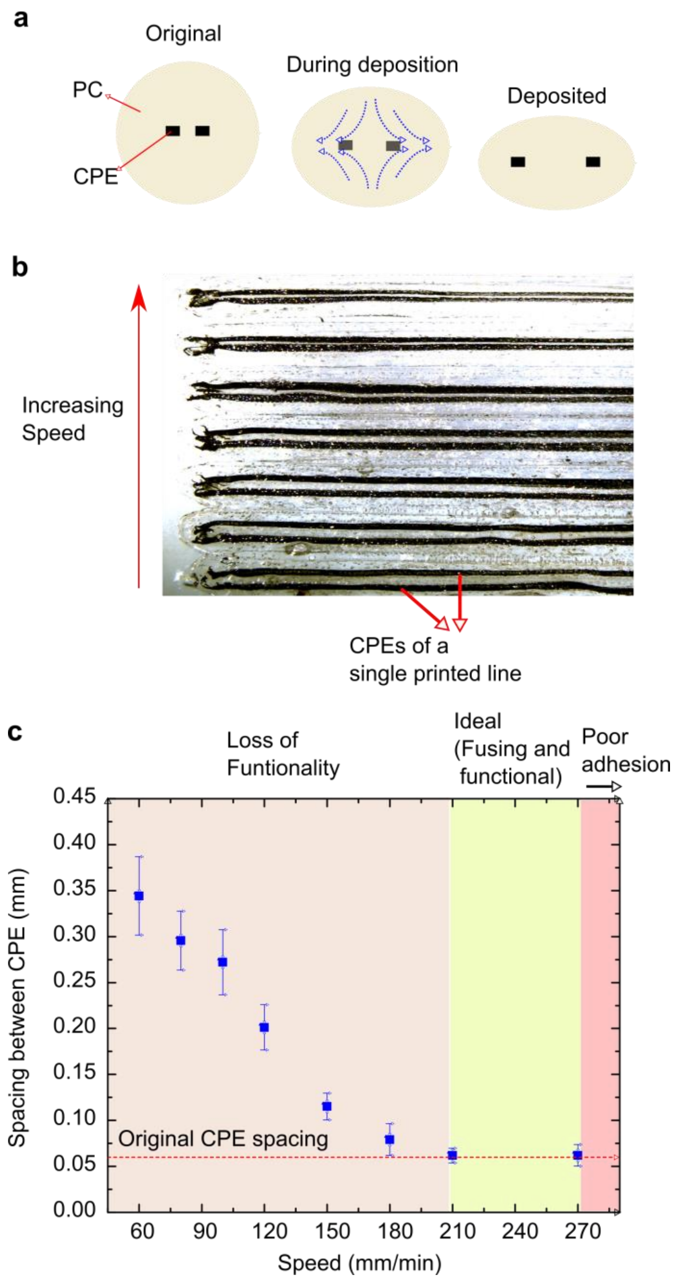

Supplementary Figure 5: Separation of materials during deposition. (a) Due to the flow of the viscoelastic matrix (PC) during wetting and deposition, the materials within the filament (such as CPE) are being pushed further apart from each other. **(b)** Optical micrograph of the printed filament with its inner structure design, containing two CPE blocks, as shown in a. The spacing between the CPE blocks decreases for increasing speed. **(c)** Plot of CPE spacing versus deposition speed. Increasing the deposition speed results in a decrease in spacing between the CPE until it eventually reach the CPE spacing of the original filament. The filaments are printed at increasing speeds and at the common hot end temperature of 340 ^{o}C. Note for the printed line at 270 mm/min, it is still able to adhere to the underlying polycarbonate print bed. The error bars are produced by calculating the standard deviation of 10 measurements of the spacings between CPE with each measurement made at different point along the printed filament.

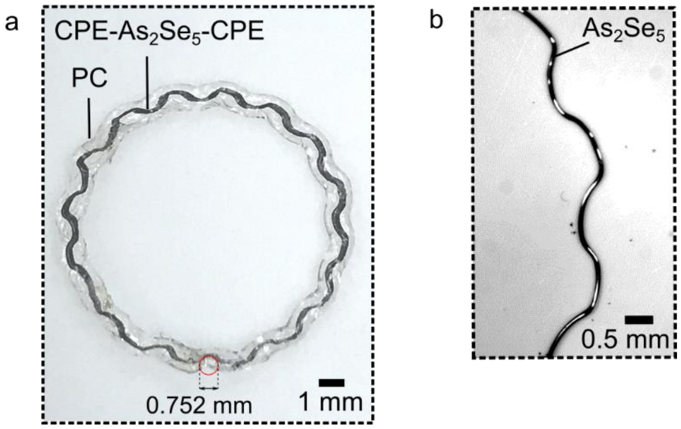

Supplementary Figure 6: High-curvature vase. (a) The vase comprises of multiple layers of printed lines, with each line making up of multiple serpentines. The functional domain (CPE-As_2Se_5-CPE) is continuous throughout these serpentines, resulting in sensing elements in sharp curves. The maximum curvature radius measured of the functional domain is 0.38 mm. **(b)** We removed the PC and CPE from the printed line to demonstrate continuity of the As_2Se_5 throughout the entire structure. Note that As_2Se_5 is a brittle, glassy material which tends to shatter and break upon bending. The continuity of As_2Se_5 preserves the photodetecting structure and performance along the kinks.

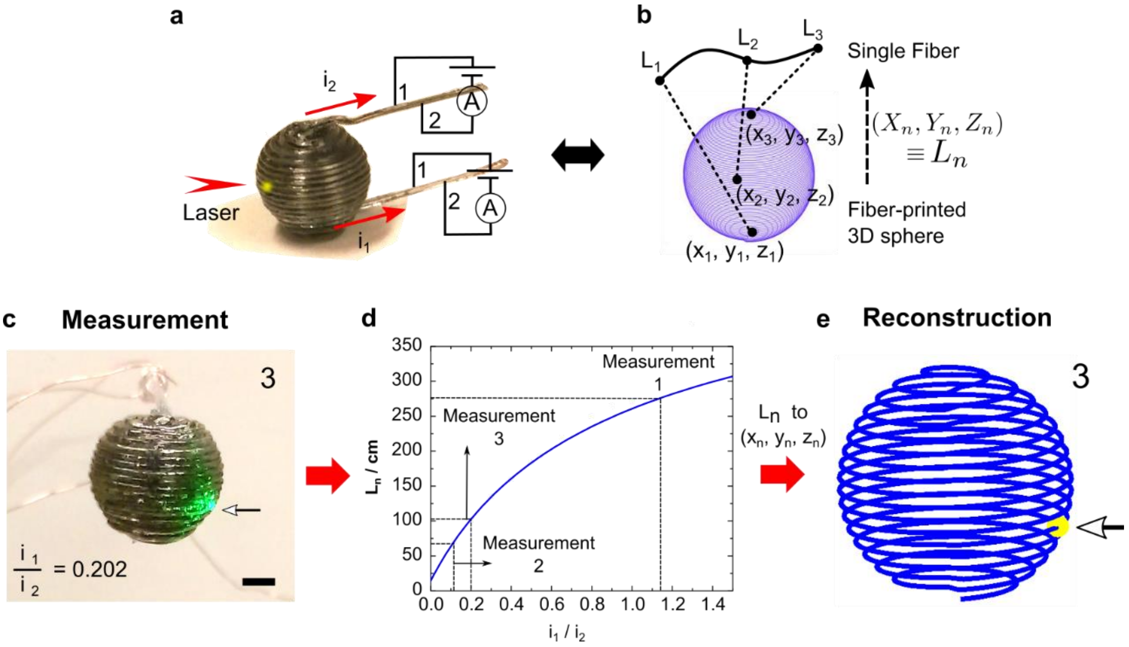

Supplementary Figure 7: Omnidirectional localized-sensing device. (a) Experimental setup to characterize localized photodetection on a printed sphere. The sphere is printed from a single continuous photodetecting filament. Electrical connections are made at both ends of the printed filament with the same voltage polarity across the electrodes (Electrode 1 and Electrode 2 are attached to positive and negative voltages, respectively). Two photocurrent values, i_1 and i_2, are collected from both ends of the 3D printed structure upon laser light impingement. **(b)** The 1-dimensional axial position L_n of the filament undergoes one-to-one mapping with the 3-dimensional (X, Y, Z) coordinates of a printed 3D macrostructure. **(c)** Laser light (arrowed) is impinged at 3 different locations on the surface of the printed sphere, labelled 1, 2 (in Fig. 4g and 4h) and 3, and the ratio of i_1/i_2 is measured for each state. (Scale bar, 2 mm). **(d)** The one-to-one relationship between the axial length L_n and the current ratio (i_1 / i_2) is derived from the axial non-uniformity voltage profile across the printed filament that provides distinct current ratio value at different points along the axial length of a printed filament[2]. The current ratios and the corresponding L_n values of the 3 different light impingement locations (1, 2 and 3) are indicated in the graph as measurement 1, 2 and 3, respectively. **(e)** MATLAB-generated reconstruction of the sphere that show the exact position of light impingement on its curved sphere surface, calculated by using the ratio of the two currents. The reconstructed positions generated in locations 1, 2 and 3 correspond closely to the actual positions of the light impingement.

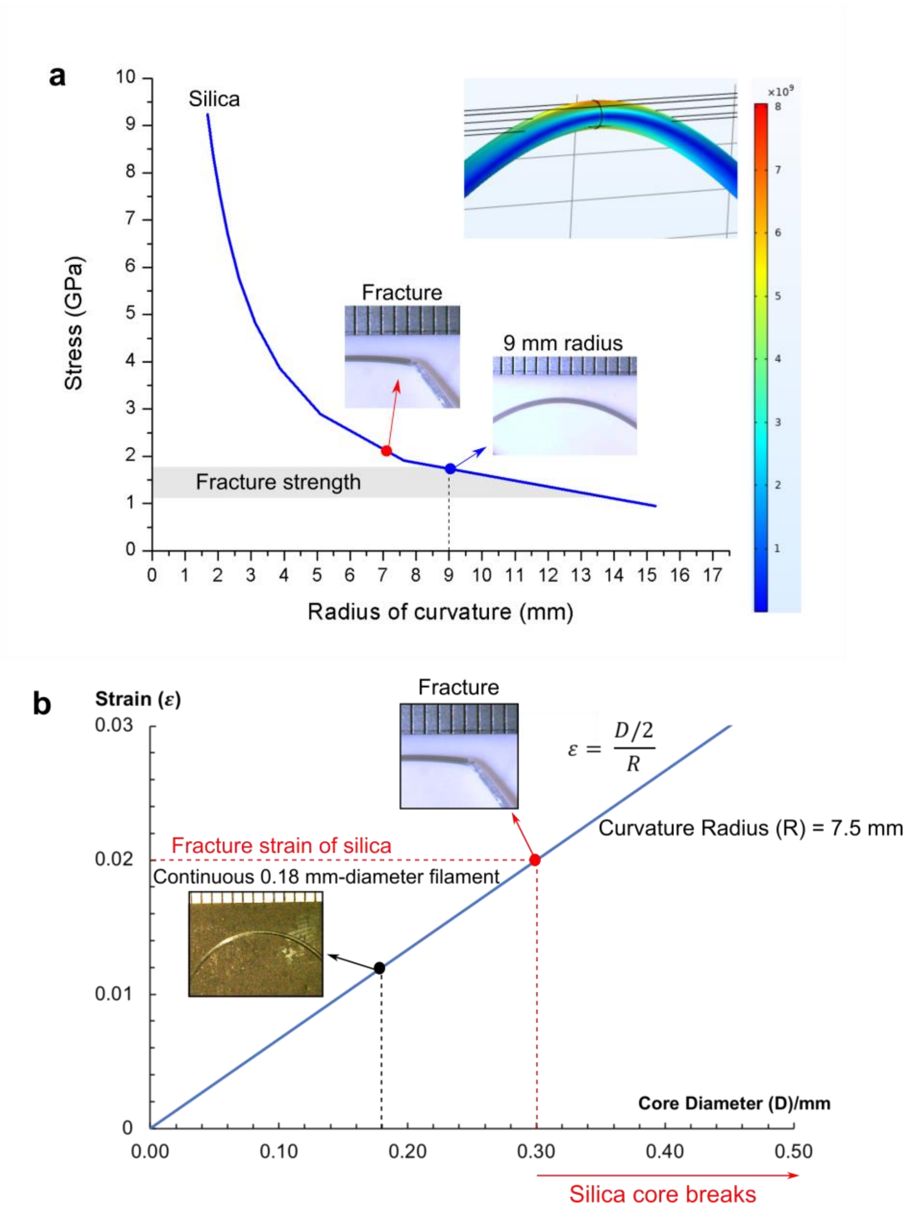

Supplementary Figure 8: Material requirements in printing high curvature turns for brittle materials. (a) Plot of stress versus curvature radius for silica which undergoes brittle fracture with no plastic deformation. This plot is obtained by measuring the maximum stress through COMSOL simulation. The simulation indicates that the silica breaks at around 8.5 mm curvature radius. The experimentally-obtained radius of curvature at fracture is 7.5 mm (See inset optical micrographs), while the theoretical elastic bending strain equation[12] $\varepsilon = D/2R$ (where ε is the bending strain, D is the core diameter, and R is the radius of curvature) gives a minimal radius of curvature of 7.5 mm. The minimum curvature radius obtained via $\varepsilon = D/2R$ corresponds closely to both the experimental and simulation results. **(b)** By using the elastic bending strain equation $\varepsilon = D/2R$, we plotted a linear line relating strain to core diameter for a curvature radius of 7.5 mm. This equation can be used to determine if a material of a specific filament core diameter breaks upon printing high curvature turns. Note that this equation is only valid[12] for materials that undergo brittle fracture, meaning fracturing of materials during the elastic limit with no plastic deformation. For the plot above, a 300 um-diameter silica filament, with a fracture strain[13] of 0.02, breaks when forming a bend with radius of curvature of 7.5 mm. However, by decreasing the core diameter lower than 300 um, e.g. 180 um, one can

form bends with the same radius of curvature and the same material without fracturing. Similarly, one can use this same elastic bending strain equation to determine the required core diameter of other functional brittle materials (group IV materials such as silicon[14] with fracture strain of 0.0055; and group III-IV materials such as GaN[15] with fracture strain of 0.031), so as to be able to print them to the required radius of curvature without fracturing.

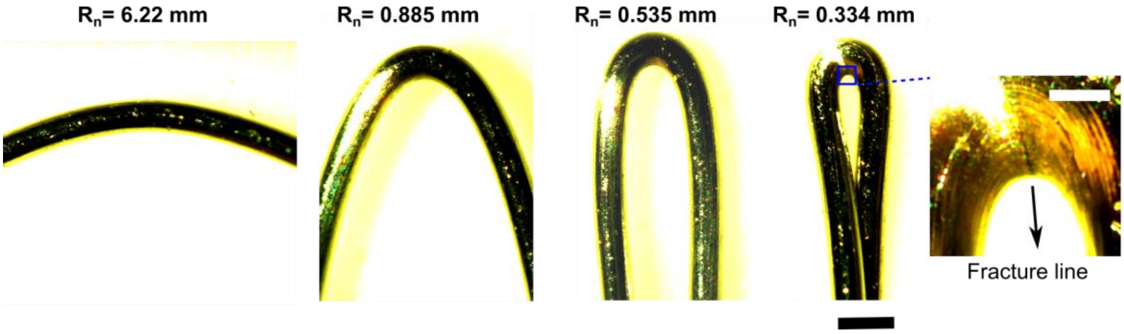

Supplementary Figure 9: Material requirements in printing high curvature turns for plastically deforming materials. Images of W wire with a diameter of 300 um with different bending curvature radii (Scale bar, 1 mm). The W wire is bent till it fractures (Scale bar for inset, 0.1 mm). The curvature radius at fracture is measured to be 0.334 mm, which makes use of the equation $R_n = \sqrt{R_i R_0}$ found in Ref. [12], where R_n is the curvature radius to the neutral axis, R_i and R_o are the inner and outer radius of curvature, respectively. For W, the minimum curvature radius cannot be predicted by the elastic bending strain equation, from which the radius is calculated to be a much higher value of 0.882 mm for a W fracture strain[16] of 0.17. This is because W plastically deforms[16] and undergoes strain hardening[17]. Nevertheless, the elastic bending strain equation provides an upper limit to the minimal curvature radius for W.

Supplementary Table 1: **A summary of the total device volume, time taken, volumetric speed, and types of device shapes formed through different 3D-device fabrication approaches.** The time is measured as the duration for constructing the 3D-device topologies, after the device constituents are already prepared. For example, for embedded 3D-print, the time taken is that for device-embedding, printing and ink curing, not considering the time for fabricating the individual discrete chips; for kirigami, the time taken is that for laser cutting or transfer printing, not considering the multiple steps for multilayer device fabrication; for manual assembly of fibers, the time taken is that of manually arranging the fibers at a precision of 0.5 mm, not considering the fiber fabrication time. (Here, we assume that the volumetric speed for fabrication scales proportionally with smaller precision. For example, for lower precision of 1 cm, the volumetric speed of fabrication will increase to ~ 80 mm^3/min); For this work, the time taken is that of printing the functional device filaments, not considering the fiber fabrication time.

3D-Device Fabrication	Device Volume	Time	Volumetric Speed (Device volume/Time)	Shape
Embedded-3D print	• Dependant on the volume of devices embedded into the 3D-structure.	• Embedding is still done manually due to the non-planar 3D architecture.[3]<br>• Curing of metallic electrodes for each print layer takes hours.[3,4,5]	~ 5.1 mm^3/min [4]	• Customizable in overall printed shape, but shapes of internal slots and channels are restricted to the geometries of embedded devices.
Kirigami	• Pop-up 2D.<br>• 3D structures are hollow. Device functionality is present only at its surface, and not within the core.<br>• Functional volume depends on planar area multiplied by thickness of device structure.<br>• Large planar area, but thickness has to be thin for laser-cutting and folding.	• For laser-cut kirigami, time is spent on laser cutting, which is proportional to device area.[6]<br>• For buckling kirigami, time is spent on transfer printing. (~ 5 hours)[7]	~ 32.4 mm^3/min [6]	• Limited to folding rules.
Manual Assembly of fibers	• Large outer area formation, but similarly the bulk of the structure is empty.[8] Density is usually small due to time constraint. Precision of assembly is in cm-scale.[8]	• Fabrication is slow especially at higher spatial precision.[9,10,11]	~ 0.2 mm^3/min [11]	• Difficulty in forming high-curvature and highly dense structures at high precision.
This work	• Large device-volume printing, demonstrated by the printed functional aeroplane wing.<br>• Fully 3-dimensional with device both at the surface and within the bulk of the structure. Print precision is 0.5 mm.	• Time is dependent on the line print speed.	• Volumetric device print speed ~ 200 mm^3/min	• Fully-Customizable.

Supplementary Notes

Supplementary Note 1: Relationship between width of light-emission and sphere size

In this note, we derived the relationship between the width of light-emission and sphere size with reference to Supplementary Fig. 1. By making use of the capacitive voltage divider[1] across the PC, ZnS, and the dielectric, the electric field across ZnS (E_{ph}) can be represented by:

$$E_{\mathrm{ph}} = \frac{V}{d_{\mathrm{ph}} + \frac{\epsilon_{\mathrm{ph}}}{\epsilon_{\mathrm{di}}} d_{\mathrm{di}} + \frac{\epsilon_{\mathrm{ph}}}{\epsilon_{\mathrm{pc}}} y_{\mathrm{pc}}} \qquad (1)$$

, where V is the input voltage difference between the BiSn and the copper, d_{ph} and ϵ_{ph} are the thickness and the dielectric constant of the ZnS phosphor, respectively, d_{di} and ϵ_{di} are the thickness and the dielectric constant of the dielectric, respectively, and finally, y_{pc} and ϵ_{pc} are the thickness of the PC (distance between circumferential point of the sphere to ZnS and the dielectric constant of PC. After rearranging the above equation, and inputting the geometric relationship between the sphere radius and y_{pc}:

$$\frac{V}{E_{\mathrm{ph}}} = d_{\mathrm{ph}} + \frac{\epsilon_{\mathrm{ph}}}{\epsilon_{\mathrm{di}}} d_{\mathrm{di}} + \frac{\epsilon_{\mathrm{ph}}}{\epsilon_{\mathrm{pc}}} \left[R - \sqrt{R^2 - x_{\mathrm{light}}{}^2}\,\right] \qquad (2)$$

Let A_{R} represents the following:

$$A_{\mathrm{R}} = d_{\mathrm{ph}} + \frac{\epsilon_{\mathrm{ph}}}{\epsilon_{\mathrm{di}}} d_{\mathrm{di}} + \frac{\epsilon_{\mathrm{ph}}}{\epsilon_{\mathrm{pc}}} R$$

Since ϵ_{di}= 1700 and ϵ_{ph}= 8.9 , $\epsilon_{\mathrm{di}} \gg \epsilon_{\mathrm{ph}}$. We thus obtain:

$$A_{\mathrm{R}} = d_{\mathrm{ph}} + \frac{\epsilon_{\mathrm{ph}}}{\epsilon_{\mathrm{pc}}} R \qquad (3)$$

Substituting Equation (3) into Equation (2), and replacing E_{ph} by $E_{\mathrm{ph,T}}$ which represents the threshold electric field at which ZnS lights up, one will obtain the following:

$$\frac{V}{E_{\mathrm{ph,T}}} = A_{\mathrm{R}} - \sqrt{R^2 - x_{\mathrm{light}}{}^2}$$

Rearranging this equation and making x_{light} the subject:

$$x_{\text{light}} = \sqrt{R^2 - (A_{\text{R}} - \frac{V}{E_{\text{ph,T}}})^2}$$

Since x_{light} only represents one side of the sphere, we should multiply by 2 to obtain the width of light-emission. This width is also dependent on the exposure value that is used to capture the images. Higher exposure results in higher measured width. Hence, we include a proportionality constant, *C*, that relates the width of light-emission with x_{light}:

$$Width\ of\ light\ emission = C\sqrt{R^2 - (d_{\text{ph}} + \frac{\epsilon_{\text{ph}}}{\epsilon_{\text{di}}} d_{\text{di}} + \frac{\epsilon_{\text{ph}}}{\epsilon_{\text{pc}}} R - \frac{V}{E_{\text{ph,T}}})^2}$$

Inputting the dielectric values of ϵ_{di}= 1700, $\epsilon_{\text{pc}} = 2.9$, and ϵ_{ph}= 8.9, the thickness values of d_{di}= 18 um, and d_{ph} = 18 um , the threshold electric field ($E_{\text{ph,T}}$) measured for this phosphor to be 5.56 x 10^6 Vm^{-1}, and the sphere radius measured through optical micrographs, one then obtain the theoretical plots in Figure 2c.

Supplementary Note 2: Approach to increase print precision

In the main text, we describe a print precision of 0.5 mm. To increase the print precision of this device-printing approach, both the diameter of the filament and the nozzle orifice have to decrease. The size of the nozzle orifice can be reduced through precise machining methods such as laser cutting or milling, while the filament diameter can be reduced by increasing the draw speed during thermal drawing of the filament. However, another contributing factor, that impedes a filament diameter smaller than 0.5 mm to be printed in this work, is attributed to filament buckling[18,19]. Filament buckling arises, in specific, from the printer infrastructure. It leads to jamming and congestion of the heated nozzle. In our printer infrastructure, feed rollers are used to push the filament through the heated nozzle. As the rollers push the filament, the filament is axially compressed along its length, leading to buckling especially as the filament diameter is smaller. To determine the smallest achievable fiber diameter such that buckling is prevented, we use the Euler Buckling equation[19]:

$$\sigma_{\mathrm{cr}} = \frac{\pi^2 E}{16(\frac{L}{D})^2 K}$$

, where σ_{cr} is the critical stress at which the filament buckles, *E* is the elastic modulus of the filament, *L* is the length between the feed rollers and the heated nozzle, *D* is the filament diameter and *K* is the corrective factor as the nozzle diameter is slightly bigger than the filament diameter. Through some re-arrangement of the above equation, one can then correlate the filament diameter, *D*, to the length, *L*:

$$D = \sqrt{\frac{16\,K\,\sigma_{\mathrm{cr}}}{\pi^2 E}} \cdot L = A \cdot L$$

, where *A* is a proportionality constant which equates to 8.33 x 10^{-4} by substituting *D* = 0.5 mm and *L* = 600 mm measured for our printer setup. From this equation, one can observe that the minimum printable filament diameter is dependent on the length, *L*, between the rollers

and the nozzle. As such, by decreasing L, the probability of buckling decreases, allowing for filament diameter much smaller than 0.5 mm to be printed.

Supplementary References

1. Shen, Y. *et al.* High energy density of polymer nanocomposites at a low electric field induced by modulation of their topological-structure, *J. Mater. Chem. A* **4**, 8359 (2016).

2. Sorin, F. *et al.* Exploiting Collective Effects of Multiple Optoelectronic Devices Integrated in a Single Fiber, *Nano Lett.* **9**, 7 (2009).

3. MacDonald, E. *et al.* 3D printing for the rapid prototyping of structural electronics. *IEEE Access* **2**, 234–242 (2014).

4. Li, J. *et al.* Hybrid additive manufacturing of 3D electronic systems. *J. Micromechanics Microengineering* **26**, (2016).

5. Lopes, A. J., MacDonald, E. & Wicker, R. B. Integrating stereolithography and direct print technologies for 3D structural electronics fabrication. *Rapid Prototyp. J.* **18**, 129–143 (2012).

6. Lamoureux, A., Lee, K., Shlian, M., Forrest, S. R. & Shtein, M. Dynamic kirigami structures for integrated solar tracking. *Nat. Commun.* **6**, (2015).

7. Lee, W. *et al.* Two-dimensional materials in functional three-dimensional architectures with applications in photodetection and imaging. *Nat. Commun.* **9**, (2018).

8. Abouraddy, A. F. *et al.* Large-scale optical-field measurements with geometric fibre constructs. *Nat. Mater.* **5**, 532–536 (2006).

9. Kato, Y., Seikai, S. & Tanifuji, T. Arc-Fusion Splicing of Single-Mode Fibers: An Apparatus with an Automatic Core-Axis Alignment Mechanism and Its Field Trial Results. *J. Light. Technol.* **2**, 442–447 (1984).

10. Kim, B., Kang, H., Kim, D. H. & Park, J. O. A flexible microassembly system based on hybrid manipulation scheme for manufacturing photonics components. *Int. J. Adv. Manuf. Technol.* **28**, 379–386 (2006).

11. Miller, A. J. *et al.* Compact cryogenic self-aligning fiber-to-detector coupling with losses below one percent. *Opt. Express* **19**, 9102 (2011).

12. Kuhn, H. & Medlin, D. *ASM Handbook, Volume 8: Mechanical Testing and Evaluation, Stress-Strain Behavior in Bending*. *ASM* (2000).

13. Biswas, A. K., Cherif, C., Hund, R.-D., Shayed, M. A. & Hossain, M. Influence of Coatings on Tensile Properties of Glass Fiber. *Mater. Sci.* **20**, (2014).

14. Sharpe, W. N., Bagdahn, J., Jackson, K. & Coles, G. Tensile testing of MEMS materials-recent progress. *J. Mater. Sci.* **38**, 4075–4079 (2003).

15. Brown, J. J. *et al.* Tensile measurement of single crystal gallium nitride nanowires on MEMS test stages. *Sensors Actuators, A Phys.* **166**, 177–186 (2011).

16. Levin, Z. S., Srivastava, A., Foley, D. C. & Hartwig, K. T. Fracture in annealed and severely deformed tungsten. *Mater. Sci. Eng. A* **734**, 244–254 (2018).

17. Scapin, M., Fichera, C., Carra, F. & Peroni, L. Experimental investigation of the behaviour of tungsten and molybdenum alloys at high strain-rate and temperature. *EPJ Web Conf.* **94**, 01021 (2015).

18. Yang, Z., Jin, L., Yan, Y. & Mei, Y. Filament breakage monitoring in fused deposition modeling using acoustic emission technique. *Sensors (Switzerland)* **18**, (2018).

19. Venkataraman, N. *et al.* Mechanical and rheological properties of feedstock material for fused deposition of ceramics and metals (FDC and FDMet) and their relationship to process performance. *Proc. Solid Free. Fabr. Symp.* 351–359 (1999).
