# Supplementary material for: Structured multimaterial filaments for 3D printing of optoelectronics
Source: Nat Commun. 2019 Sep 5;10:4010. doi: 10.1038/s41467-019-11986-0 (PMC6728390; doi:10.1038/s41467-019-11986-0)
Supplement: Supplementary file 3 — Description of Additional Supplementary Files [file 41467_2019_11986_MOESM3_ESM.pdf]

Description of Additional Supplementary Files

File Name: Supplementary Movie 1
Description: Multimaterial Filament Printing of Photodetecting pyramid (Time x15) through filament surface heating. The modified nozzle consists of a hot end which is heated by the red hot nichrome wire. The base width of the pyramid is 1.55 cm.
